# A Low-Cost Wheat Bran Medium for Biodegradation of the Benzidine-Based Carcinogenic Dye Trypan Blue Using a Microbial Consortium

**DOI:** 10.3390/ijerph120403480

**Published:** 2015-03-25

**Authors:** Harshad Lade, Avinash Kadam, Diby Paul, Sanjay Govindwar

**Affiliations:** 1 Department of Environmental Engineering, Konkuk University, Seoul 143-701, Korea; E-Mail: harshadlade@gmail.com; 2 Department of Environmental Engineering, Kyungpook National University, Daegu 702-701, Korea; E-Mail: avikadam2010@gmail.com; 3 Department of Biochemistry, Shivaji University Kolhapur 416004, India

**Keywords:** wheat bran, benzidine-based dyes, Trypan Blue, mutagenic, microbial consortium, decolorization, biodegradation, detoxification

## Abstract

Environmental release of benzidine-based dyes is a matter of health concern. Here, a microbial consortium was enriched from textile dye contaminated soils and investigated for biodegradation of the carcinogenic benzidine-based dye Trypan Blue using wheat bran (WB) as growth medium. The PCR-DGGE analysis of enriched microbial consortium revealed the presence of 15 different bacteria. Decolorization studies suggested that the microbial consortium has high metabolic activity towards Trypan Blue as complete removal of 50 mg∙L^−1^ dye was observed within 24 h at 30 ± 0.2 °C and pH 7. Significant reduction in TOC (64%) and COD (88%) of dye decolorized broths confirmed mineralization. Induction in azoreductase (500%), NADH-DCIP reductase (264%) and laccase (275%) proved enzymatic decolorization of dye. HPLC analysis of dye decolorized products showed the formation of six metabolites while the FTIR spectrum indicated removal of diazo bonds at 1612.30 and 1581.34 cm^−1^. The proposed dye degradation pathway based on GC-MS and enzyme analysis suggested the formation of two low molecular weight intermediates. Phytotoxicity and acute toxicity studies revealed the less toxic nature of the dye degradation products. These results provide experimental evidence for the utilization of agricultural waste as a novel low-cost growth medium for biodegradation of benzidine-based dyes, and suggested the potential of the microbial consortium in detoxification.

## 1. Introduction

The U.S. Environmental Protection Agency (EPA) has identified an initial list of widely recognized benzidine-based and benzidine congener-based dyes for action plan development due to their human toxicity, recalcitrance and bioaccumulative characteristics [1]. There is a well-established concern for the potential human carcinogenic effects presented by exposure to benzidine-based dyes [1]. These dyes get broken down to certain aromatic amines of concern such as benzidines, which are known as possible carcinogens or genotoxicants [2]. It is well-known that benzidine and its congeners react with DNA which results in mutations [3]. There are many reports on the mutagenicity of benzidine-based dyes and their congeners [4,5,6]. In addition, their degradation metabolites, e.g. aromatic amines, are reported as toxic, mutagenic as well as carcinogenic, which may pose additional health hazards to humans [7,8].

Benzidine-based and congener-based dyes are widely used in the production of textiles, paints, printing inks, leather colorings, paper making, pharmaceuticals and food industries [1]. The excessive use of such dyes at industrial levels leads to their release to the environment through waste disposal and thus may contaminate river waters, sediments, sludges and drinking waters. A systematic backtracking of the flow of wastewater from textile processing industries within the European Union has identified several benzidine-based mutagenic dyes in exposed sites [9]. Some of such dyes as well as their degradation metabolites may pass through the food chain from soil or water and lead to serious ecological and human health effects. Examples of benzidine-based dyes include the blue acidic azo dye Trypan Blue, which is extensively used in the textile, food and paint industries for dyeing silk, cotton, wool, nylon and also for coloring oil, waxes, varnish, plastics, *etc.* Release of Trypan Blue in the environment creates serious human health problems as it is known for its enhanced mutagenicity and high recalcitrance towards microbial degradation because of its fused aromatic ring structure [10]. Trypan Blue has been reported as carcinogenic in rats and its administration produced reticulum-cell sarcomas of the liver and fibrosarcomas at the site of injection [11]. Thus, there is a significant interest in developing effective treatment technologies for the complete removal of benzidine-based dyes from environmental sinks as their discharge poses mutagenic and/or carcinogenic risks to mammals and/or humans.

Removal of dyes from environmental sinks can be achieved through different physicochemical and biological processes. The physicochemical methods applied include membrane separations, photocatalysis, sonication, irradiation, photochemical process, electrochemical oxidation, ion exchange, activated carbon adsorption, coagulation/flocculation, ozonation and Fenton processes [12,13,14]. A widely used commercial chemical method of dye removal is coagulation which rapidly transfers dyes from the liquid to the solid phase but has several drawbacks such the inability to remove dyes completely, the high cost of the chemicals used, generation of hazardous secondary wastes and its disposal problem [15,16]. Another physical method like adsorption on low cost adsorbents like fly ash, activated carbon and/or agricultural waste is an inexpensive and commonly used process for removal of dyes from industrial wastewaters; but the release of adsorbed dyes is a major issue of concern as these remain in the environment for long time [13,17]. Advanced oxidation processes generate the second most powerful oxidant, the OH free radical, to oxidize dyes ultimately to CO_2_ and H_2_O, but these processes are also not sufficient to completely remove dyes and are known to generate secondary wastes which are reported as toxic or even more harmful than the parent dyes [16,18,19,20]. Overall, each physicochemical method has its own advantages and drawbacks. It is thus necessary to use an alternative eco-friendly and cost effective treatment method which can remove the dyes as well as detoxify them. Thus, bioremediation has been suggested as a promising method which not only potentially mineralizes dye molecules into CO_2_ and H_2_O, but also generates low amounts of sludge [21].

Over the past decade, several bacteria, fungi, algae, lichen, plants as well as their consortia have been shown able to degrade structurally different hazardous dyes [22,23,24,25,26,27,28]. Among these promising biological systems, bacteria have attracted the attention of the scientific community because of their short life cycle, less secondary pollution and the fact they can grow on various substrates. Indeed, most research to date has been conducted by using defined growth media such as nutrient broth for cultivating bacteria, which is not affordable to use at commercial levels. Therefore, research is needed to find alternative carbon- and nitrogen-containing substrates that can support bacterial growth and are also easily available at low cost. The possible use of agricultural waste as a low cost carbon and nitrogen source should not be neglected. Several crop biomass such as rice bran, sugarcane bagasse, wheat bran, *etc.* are generated every year in million ton amounts as “agricultural waste”, easily available at low cost and they are known to contain high amounts of carbon and nitrogen [29,30]. Recently, agricultural waste rice bran, yeast biomass and sugarcane bagasse were reported as promising growth media for the removal of dyes from textile wastewaters in solid state fermentation (SSF) processes [30,31,32]. However, at present submerged fermentation technology has well-developed protocols for wastewater treatments, while at the same time SSF is at the development stage for field level applications [33]. Hence it is quite beneficial to use submerged fermentation conditions for wastewater treatment using agricultural wastes as growth medium. As a representative of agricultural waste, wheat bran (WB) was evaluated as a growth medium in this study because it is available at low cost and contains a combination of carbohydrates, proteins, amino acids and trace elements [34]. In addition, WB has been reported to be good a growth medium for carboxymethyl cellulose production by *Aspergillus flavus*, cellulose production by *Paenibacillus terrae ME27-1* and hydrolytic enzymes production by *Bacillus megatherium* [34,35,36].

The overall aim of this research was therefore to enrich a microbial consortium having the ability to degrade and completely detoxify the benzidine-based carcinogenic dye Trypan Blue using agricultural waste WB as growth medium, to provide further understanding of the mechanisms involved in dye mineralization and also to indicate possible applicationa of agricultural waste as a low cost growth medium for bioremediation of hazardous dyes. In this work, a microbial consortium from dye contaminated soils.capable of decolorizing the benzidine-based dye Trypan Blue was enriched in WB medium. The bacterial diversity of the enriched consortium was investigated by PCR-DGGE analysis. The decolorization experiments were performed using wheat bran as growth medium under submerged fermentation conditions. Mineralization of the dye was determined by the reduction in TOC and COD values while the role of oxidoreductive enzymes in the biotransformation process was analyzed by standard assays. Degradation of the dye and formation of metabolites was confirmed by HPLC, FTIR and GC-MS analysis. Additionally, a possible pathway for the degradation of Trypan Blue dye by the microbial consortium was proposed based on the enzyme activities and analytical studies. Detoxification of dye degraded products was assessed by phytotoxicity and acute toxicity tests.

## 2. Materials and Methods

### 2.1. Chemicals and Dye Used

**Figure 1:**
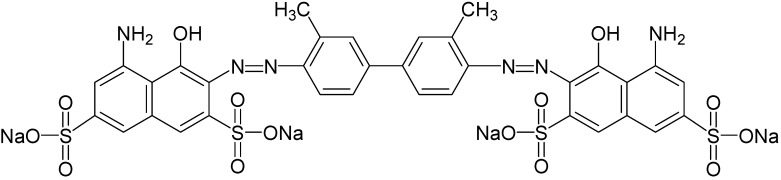
Structure of the benzidine-based azo dye Trypan Blue.

2,2’-Azino-bis (3-ethylbenzothiazoline-6-sulfonic acid) (ABTS), veratryl alcohol, catechol, veratryl alcohol, methyl red, nicotinamide adenine dinucleotide (NADH) and dichlorophenol indophenols (DCIP) were procured from HiMedia Laboratories Pvt. Ltd. (Mumbai, India). Chloranil, dimethylformamide (DMF), aniline-2-sulfonic acid and all other chemicals were obtained from Sigma-Aldrich (St. Louis, MO, USA). All the chemicals used in this study were of high purity (>98%). Trypan Blue (CAS No. 72-57-1; CI No. 23850; CI Name = Direct Blue 14; Molecular Formula = C_34_H_24_N_6_O_14_S_4_Na_4_; Molecular Weight = 960.81 g∙mol^−1^; λmax 660 nm); λmax 660 nm), a water-soluble azo dye was procured from Sigma-Aldrich and used as received. Its chemical structure is shown in Figure 1. Unless otherwise stated, the working concentration of dye Trypan Blue was 50 mg∙L^−l^.

### 2.2. Wheat Bran Medium

Agricultural waste wheat bran was obtained from a local wholesalers shop at Kolhapur (MS, India). It was passed through 50-mesh sieve and dried in an oven at 80 °C ± 1 °C until the weight was constant. For decolorization studies, five gram of dried wheat bran was boiled in 100 mL of distilled water for 15 minutes and the extract was separated by filtration through Whatman grade no. 1 filter paper. The resulting clear filtrate was then made up to a total volume of 100 mL with distilled water, the pH was adjusted to 7.0, it was autoclaved for 15 min at 121 °C and used as WB medium. Total sugars content in the WB medium was estimated by the anthrone method using glucose as a standard [37]; while protein concentration was quantified by Lowry’s method with bovine serum albumin as a standard [38].

### 2.3. Enrichment of the Trypan Blue Decolorizing Microbial Consortium

A soil sample of approximately 100.0 gm was collected from the surface layer (5–10 cm) of dye contaminated areas at Mahesh Textile Processers (Ichalkarangi, Maharashtra, India). The sample was packed in an air tight polythene re-sealable bag, kept in an ice box, transported to laboratory and stored in a refrigerator for further use. The microbial consortium capable of decolorizing Trypan Blue dye was enriched by inoculating 1.0 gm of dye contaminated soils in a 250 mL Erlenmeyer flask containing 100 mL of WB medium supplemented with 25 mg∙L^−l^ of dye. The inoculated flask was allowed to enrich under microaerophilic incubation conditions (i.e. no aeration and agitation) at 30 ± 0.2 °C for a week and further acclimatized consecutively until it showed complete decolorization of dye under the same conditions. The developed consortium was then cultured in WB medium containing 25 mg∙L^−l^ of dye Trypan Blue to retain its decolorization activity and stored at 4 ± 1 °C for two months use and in glycerol (20%, v/v) at ‒80 ± 1 °C for long term use.

### 2.4. Microbial Diversity Analysis

The polymerase chain reaction-denaturing gradient gel electrophoresis (PCR-DGGE) technique was applied to investigate the bacterial diversity present in the developed microbial consortium. Initially, the consortium was grown in WB medium for 24 h at 30 ± 0.2 °C under microaerophilic conditions. In addition, it was also grown in the presence of the dye Trypan Blue (50 mg∙L^−l^) to investigate the changes in bacterial community after dye exposure. The genomic DNA from enriched cultures was extracted as previously described and subjected to PCR amplification of 16S rRNA genes [39].

PCR amplification was performed using the forward primer RDB1-GC clamped (F58 CGCCGCC GCGCCCCGCGCCCGGCCCGCCGCCGCGGCCGCAGTTTGATCCTGGCTCA) and reverse primer RDB2 (GGACTACCAGGGTATCTAAT). The PCR reaction mixture contained a total volume of 50 μL having 1 × PCR buffer, 1 nM of dNTPs, 1 unit Taq DNA polymerase, 2 mM MgSO_4_, 2 μL of template DNA and 0.25 pM of forward and reverse primers [13]. The temperature cycling conditions were as follows: initial denaturation for 5 min at 95 °C followed by 35 cycles of 15 s at 95 °C, 15 s at 50 °C, and 15 s at 72 °C followed by 10 min final extension at 72 °C. The amplified products were purified by PureLink PCR purification kit (Invitrogen, Bedford, MA, USA).

DGGE analysis was performed using a D-Code System (Bio-Rad Laboratories, Singapore) maintained at a constant temperature of 60 °C in 1x TAE buffer. PCR amplicons were loaded onto 8% (w/v) polyacrylamide gels (37.5:1, acrylamide/bisacrylamide) using a denaturing gradient ranging from 30% to 55% denaturant (100% denaturant is defined as 7 M urea and 40% (v/v) formamide). The electrophoresis was run at 75 V for 16 h, stained with silver nitrate and visualized [13,40]. Silver stained DGGE gel images were captured using Uvitec Gel Doc system (UVitec Ltd., Cambridge, UK).

### 2.5. Pre-enrichment of the Microbial Consortium

The developed microbial consortium stock culture was routinely pre-enrichment in WB medium. One hundred µL of 48 h old microbial consortium culture was inoculated in a 250 mL Erlenmeyer flask containing 100 mL WB medium and incubated at 30 ± 0.2 °C for 24 h under shaking condition (120 rpm). This overnight-grown culture was then used as inoculum for further dye degradation studies. Unless otherwise specified, these pre-enrichment conditions were maintained throughout the experiments.

### 2.6. Decolorization Studies

All decolorization experiments were performed in 250 mL Erlenmeyer flasks containing 100 mL of pre-enriched microbial consortium. Initially, 50 mg∙L^−1^ of Trypan Blue dye was added into the pre-enriched culture flaks and kept under microaerophilic and aerobic (shaking at 120 rpm) incubation conditions at 30 °C ± 0.2 °C. Aliquots of 2 mL were withdrawn from the experimental and control media at regular intervals of 8 h till 24 h and the cell mass was harvested by adding an equal volume of ethyl acetate followed by centrifugation (7500 × *g* for 20 min, 4 ± 0.2 °C) to obtain a clear supernatant. Decolorization of the dye Trypan Blue was measured spectrophotometrically at a λ_max_ of 660 nm (U-2800 UV-vis spectrophotometer, Hitachi, Tokyo, Japan). Pre-enriched microbial consortium culture without dye was considered as the biotic control and tested under the same conditions. Uninoculated medium containing 50 mg∙L^−1^ of dye served as the abiotic control. Unless otherwise specified, all the experiments were performed in three sets. The percent decolorization was calculated using the formula:

(1) $$\text{Decolorization }(\%)=\frac{{\text{Initial absorbance}}_{\left(\text{0 h}\right)}-{\text{ Observed absorbance after incubation}}_{\left(\text{t}\right)}}{{\text{Initial absorbance}}_{\left(\text{0 h}\right)}}\times 100$$

### 2.7. Optimization of Decolorization Parameters

In order to develop an efficient decolorization process, the optimization of various environmental parameters was carried out by a one parameter at a time approach in WB medium [27]. This includes initial pH of the pre-enriched culture medium (5–10), incubation temperature (20, 30, 37, 40 and 50 ± 0.2 °C) and various dye concentration (25–100 mg∙L^−1^).

### 2.8. Determination of Aromatic Amines, TOC and COD

The aromatic amines formed during decolorization process were estimated spectrophotometrically as described earlier [41]. Aliquots of 2 mL were withdrawn from the experimental and control media at regular intervals of 8 h and freeze-dried in an Upright Freeze Dryer (FDU5003/8603, Operon Co. Ltd., Seoul, Republic of Korea). Five milligrams of freeze-dried samples were dissolved in 5 mL of a 0.4% chloranil solution in DMF, heated at 100 ± 1 °C for 5 min and absorbance was measured at 560 nm. A calibration curve of aniline-2-sulfonic acid as a model amine product of azo dyes reduction was prepared. The concentration of amines formed in sample were calculated from standard curve and mentioned in mM.

Dye mineralization was confirmed by analyzing the total organic carbon (TOC) and chemical oxygen demand (COD) of treated dye medium. TOC was estimated using an automated TOC analyzer (Hach DR 2700 spectrophotometer, Hach Co., Loveland, CO, USA), while the COD was measured according to standard methods [42]. For this, experimental and control medium were taken, centrifuged (7500 × *g* for 15 min, 4 ± 0.2 °C), supernatant collected, passed through 0.45 µm pore size filter to remove bacterial cell debris and analyzed. The removal ratio of TOC was calculated using the formula:

(2) $$\text{TOC removal ratio}(\%)=\frac{{\text{Initial TOC}}_{\left(\text{0 h}\right)}-{\text{ Observed TOC}}_{\left(\text{t}\right)}}{{\text{Initial TOC}}_{\left(\text{0 h}\right)}}\times 100$$

where, TOC_(0 h)_ and TOC_(t)_ are the initial TOC value at (0 h) and the TOC value after particular decolorization time (t), respectively.

### 2.9. Enzyme Studies

Microbial consortium was grown in WB medium amended with Trypan Blue (50 mg∙L^−1^) at 30 ± 0.2 °C for 24 h. Cell biomass was harvested by centrifugation (7500 × *g* for 15 min, 4 ± 0.2 °C). The harvested cells were suspended in potassium phosphate buffer (50 mM, pH 7.4) and lysed using Sonics-vibracell VCX130 ultrasonic cell distrupter (Sonics & Materials Inc., Newtown, CT, USA) while maintained at 4 ± 0.2 °C keeping sonifier output at 40% amplitude and seven strokes each of 30 s with 2 min rest. The processed homogenate was again centrifuged and supernatant was used as source of crude enzymes. Similar procedures were followed for extraction of enzymes from control medium.

Activities of oxidative enzymes laccase, tyrosinase and veratryl alcohol oxidase as well as reductive enzymes azoreductase and ADH-DCIP reductase were assayed using a UV-vis spectrophotometer. Laccase activity was estimated by monitoring the increase in optical density at 420 nm (ƹ_420_nm = 36,000 (M∙cm)^−1^) due to the oxidation of ABTS in 0.1 M acetate buffer (pH 4.9) [43]. Tyrosinase activity was measured at 495 nm using the method described by Zhang and Flurkey [44]. Veratryl alcohol oxidase activity was determined by monitoring the formation of veratraldehyde at 310 nm (ƹ_310_nm= 9300 (M∙cm)^−1^ [45]. Azoreductase activity was measured by monitoring the reduction in concentration of Methyl Red at 430 nm (ƹ_430_nm = 23,360 (M∙cm)^−1^) [46]. NADPH-DCIP reductase activity was determined at 590 nm (19 mM^−1^∙cm^−1^) using a procedure reported earlier [47]. Proteins were determined by Lowry *et al.* method with bovine serum albumin as standard [38]. All assays were performed under ambient conditions and in triplicates.

### 2.10. Biodegradation Studies

Extraction of metabolites after decolorization of Trypan Blue (50 mg∙L^−1^) by the microbial consortium was carried out as described earlier with some modifications [48]. Briefly, 100 mL of sample was taken and harvested by centrifugation (10,000 × *g* for 20 min, 4 ± 0.2 °C) to obtain cell-free supernatant. Equal volume of ethyl acetate was added into the supernatant, mixed vigorously, organic layer separated and dried in a SpeedVac (Thermo Fisher Scientific Inc., Waltham, MA, USA). These remained metabolites were dissolved in 3 mL methanol, passed through a 0.45 µm cellulose acetate filter, reduced to 250 µL by evaporation and used for HPLC, FTIR and GC-MS analysis.

High performance liquid chromatography (HPLC) was performed on an Agilent 1200 HPLC system (Agilent, Santa Clara, CA, USA) equipped with a ZORBAX Eclipse XDB-C18 column (4.6 mm × 150 mm, 5 µm particle size) kept at 25 °C. Five µL of dye and its decolorized extract were injected into the column. The elution was carried out using isocratic mobile phase of acetonitrile-methanol (60:40) with a flow rate of 0.5 mL min^−1^ for 20 min while the UV detector was set at 210 nm. Fourier transform infrared spectra (FTIR) of the dye and its decolorized extract were collected using a Spectrum One spectrometer in the mid IR region of 400–4000 cm^−1^ with 16 scan speed (PerkinElmer, Branford, CT, USA). The samples were mixed with ACS reagent grade potassium bromide (5:95), pellets were made, fixed in sample holder and analyzed. Gas chromatography coupled with mass spectroscopy (GC-MS) analysis of metabolites was performed using a QP2010 instrument (Shimadzu Corporation, Kyoto, Japan) as mentioned previously with some modifications [49]. Gas chromatography was performed in temperature programming mode with Resteck column (0.25 mm × 30 m long) while ionization voltage set at 70 eV. The initial column temperature was maintained at 40 °C for 4 min and increased linearly at 10 °C per min to 290 °C and held for 4 min. Injection port temperature was 250 °C and mass interface was maintained at 220 °C. Helium was used as carrier gas at a flow rate of 1 mL min^−1^ for 30 min run time. Intermediates were identified on the basis of their mass spectra and NIST library data [50].

### 2.11. Toxicity Studies

Risk assessment of the dye Trypan Blue and its ethyl acetate extracted degradation products was performed by phytotoxicity and acute toxicity assays. The phytotoxicity test was performed at room temperature on two kinds of agriculturally important crops: *Sorghum vulgare* (monocot) and *Phaseolus mungo* (dicot) as mentioned earlier [27]. Ten seeds of each crop were daily irrigated with 10 mL each of Trypan Blue (50 mg∙L^−1^), its degradation metabolites (50 mg∙L^−1^) and distilled water as control. After 13 days, the length of shoot, root and seed germination was recorded. The percent of germination was calculated using the formula:

(3) $$\text{Germination }(\%)=\frac{\text{No. of seeds germinated}}{\text{No. of seeds sowed}}\times 100$$

Acute toxicity was determined on the freshwater organism *Daphnia magna* as described previously [51,52]. The Trypan Blue dye (50 mg∙L^−1^) medium decolorized using the microbial consortium was centrifuged (7500 × *g* for 20 min, 4 ± 0.2 °C), the supernatant was separated and sterilized by passing through a 0.45 µm pore size filter. The resulting clear filtrate was then used to perform sensitivity tests using 24 h old neonates. The assay was initiated by adding five neonates into each Erlenmeyer flask containing 25%, 50%, 75%, 100% dilution of filtrate. The flasks were kept at 20 ± 0.2 °C for 48 h in the absence of light and numbers of immobile organisms were counted after exposing to light for 20 seconds. Assay was performed in triplicate with distilled water as control.

### 2.12. Data Analysis

All the results reported are means of three replicates. Basic statistical analyses were performed using the software SPSS 17.0 (SPSS, Chicago, IL, USA). Significance was analyzed using an ANOVA with Tukey-Kramer multiple comparison test.

## 3. Results and Discussion

### 3.1. Microbial Diversity Analysis

**Figure 2:**
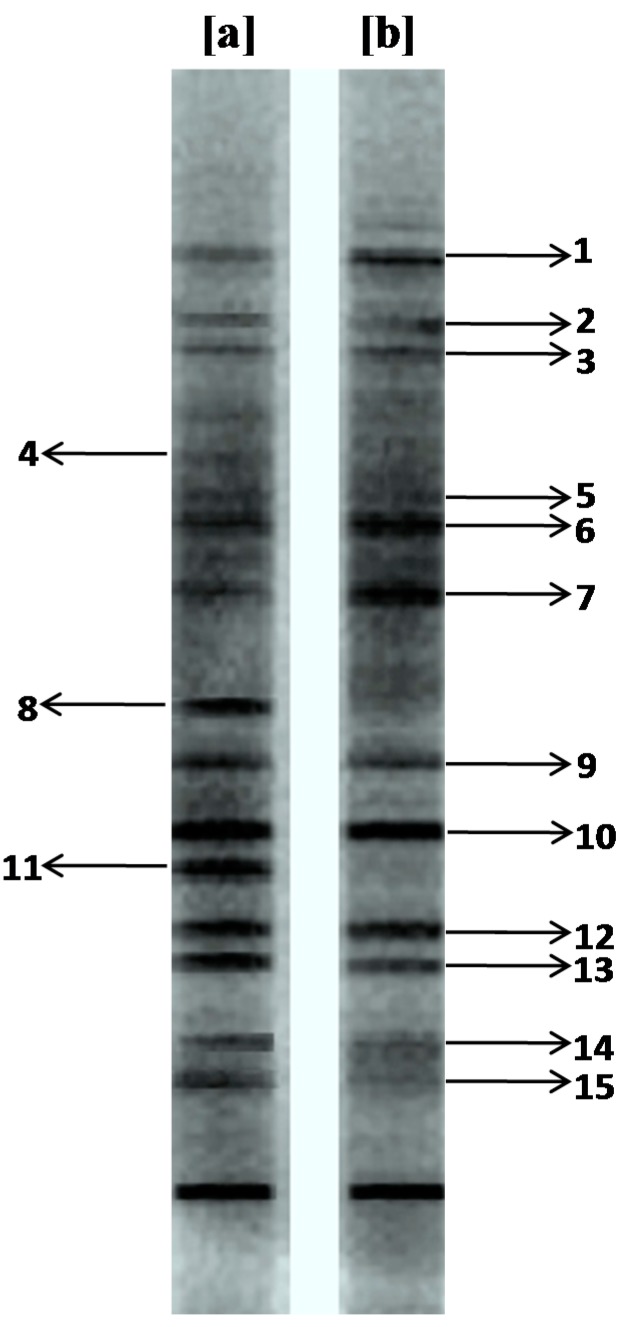
DGGE pattern of the PCR amplified 16S rDNA genes of enriched microbial consortium (**a**) before and (**b**) after decolorization of dye Trypan Blue (50 mg∙L^−1^).

PCR-DGGE, a well-established fingerprinting technique, was used to investigate the bacterial diversity of the enriched consortium, which provided meaningful information on changes in the bacterial community composition after decolorization of the dye. This technique separates the PCR amplified DNA of the same size but different sequences and thus each band obtained on a gel indicates the presence of an individual bacterium. PCR-DGGE was recently introduced in the field of molecular microbial ecology to analyze the diversity of bacterial populations [53,54]. In present study, the primary aim of the bacterial diversity analysis was to check the ability of the bacteria making up the enriched consortium to tolerate the dye Trypan Blue. Result of the DGGE analysis showed 15 distinctive bands suggesting the presence of 15 different bacteria in the enriched consortium (Figure 2a).

However, the microbial diversity analysis after exposure to the dye Trypan Blue at 50 mg∙L^−1^ concentration revealed that some bands such as 1, 2, 3, 5, 6, 7, 9, 10, 12, 13, 14 and 15 seemed to be stable in dye decolorized broth while other bands like 4, 8 and 11 detected in the enriched microbial consortium disappeared (Figure 2b). Moreover, compared with the bands in the enriched microbial consortium before use for dye decolorization, some bands such as 1, 6 and 7 became more pronounced in the dye decolorized broth. Similar observations were reported by Joshi et al. [40], where exposure of a soil bacterial population to the diazo dye Congo Red affected its community structure. As shown in Figure 2, the dye decolorized broth had a very similar bacterial community as the original enriched microbial consortium. These observations implied that most of the bacteria present in the enriched consortium have a significant ability to tolerate and/or degrade the dye Trypan Blue at 50 mg∙L^−1^ concentration. This might be due to the higher presence of textile dyestuffs in the collected soils based on the fact that continuous dyestuff amendment could enhance the microbial ability to tolerate and/or degrade dyes.

### 3.2. Decolorization of Trypan Blue

**Figure 3:**
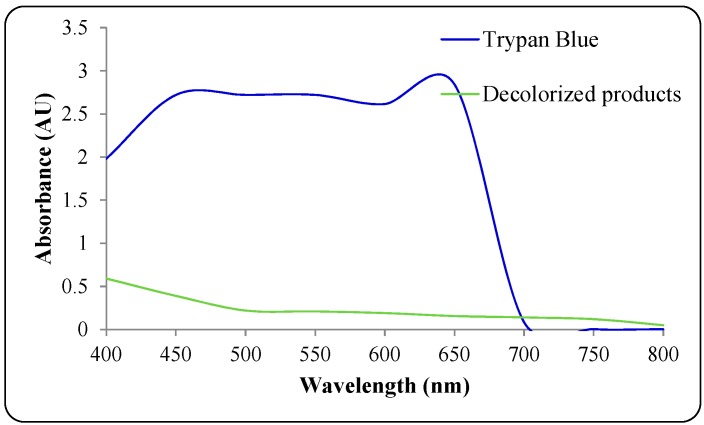
UV-visible overlay spectra of dye Trypan Blue and its ethyl acetate extracted decolorized products after 24 h treatment with microbial consortium.

Agricultural wastes are inexhaustible, non-hazardous, easily available and considered as an alternative source of nutrients to defined microbial growth media. Several agricultural wastes including rice bran, yeast biomass and sugarcane bagasse have been used previously as growth substrates for biodegradation of hazardous dyes in SSF processes [30,31,32]. In addition, wheat bran a widely available agricultural residue has been reported as a growth medium for low cost production of industrially important enzymes, but ignored in dyes degradation studies [36]. Thus, there is great possibility to use wheat bran as growth medium for the biodegradation of hazardous dyes and make the process cost effective. In the present study, wheat bran was pretreated for extraction of carbon and nitrogen sources so as to make a growth medium for bacterial cultivation and further degradation of dyes. The biochemical analysis of WB medium showed 118 mg of protein and 486 mg of total sugars. Wheat bran has been reported to contain good amounts of carbohydrates, proteins, amino acids and elements [34]. Initially, submerged culture studies on decolorization of the dye Trypan Blue (50 mg∙L^−1^) by the microbial consortium were performed in WB medium under microaerophilic conditions. UV-Vis spectrophotometric analysis of the dye and its decolorized products indicate a high decrease in the absorbance for the microbial consortium-treated medium within 24 h, suggesting dye decolorization (Figure 3). This preliminary result suggests that low cost wheat bran could be used as a growth medium for the decolorization of benzidine-based dyes. Decolorization of Trypan Blue by the self-immobilizing fungal biomass of *Pycnoporus sanguineus* has been previously reported in defined glucose-yeast extract-malt extract-peptone medium [55]. Moreover, an individual culture of thermophilic fungus, *Thermomucor indicae-seudaticae* was shown to degrade an azo-anthraquinone dye mixture containing Trypan Blue within 6 days in potato dextrose broth [56]. However, biodegradation by a single culture is known to take longer time, require defined media for growth, degrade particular dyes only and to also lead to the formation of toxic aromatic amines; while biodegradation by consortium cultures occurs faster without producing toxic amines and they also possess higher dye concentration tolerance [27]. Additionally, Trypan Blue is known to be a sturdy dye with high recalcitrance towards biological degradation [56]. Hence, to overcome the limitations of single culture, a microbial consortium consisting of 15 distinctive bacteria as determined by DGGE analysis has been investigated for the degradation and detoxification of the diazo dye Trypan Blue in present study.

**Figure 4:**
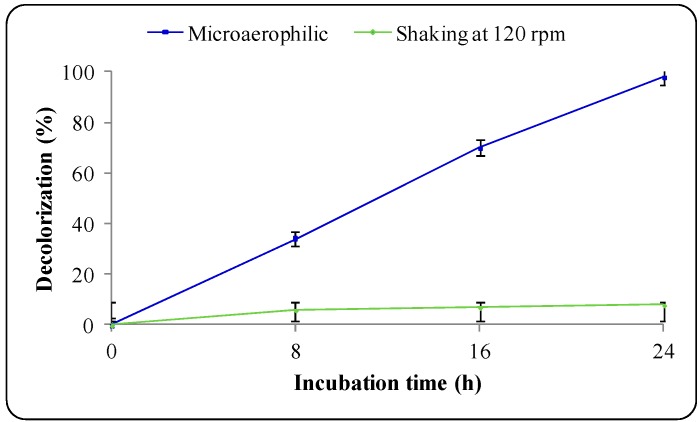
Effect of microaerophilic and aerobic (shaking at 120 rpm) conditions on decolorization of dye Trypan Blue (50 mg∙L^−1^) by microbial consortium at 30 ± 0.2 °C, pH 7.0.

Molecular oxygen is known to play an important role in the decolorization of dyes. This perception is been well supported in the present work, as aerobic incubation has an inhibitory effect on Trypan Blue decolorization whereby only 12% dye removal was observed within 24 h (Figure 4). On the contrary, almost 99% decolorization was observed in 24 h under microaerophilic conditions.

These results suggest that the microbial consortium is able to decolorize the dye completely only under microaerophilic condition. This is in agreement with previous report where aerobic conditions are known to inhibit decolorization of azo dyes primarily due to the competition in the oxidation of reduced electron carriers (e.g., NADH) with either oxygen or azo groups as the electron receptor [57]. The inhibition of Trypan Blue decolorization under aerobic conditions might be due to the the azo nature of the dye. It is stated that under aerobic conditions azo dyes are resistant to breakdown by bacteria [58]. Similarly, Phugare *et al*. during their study on the biodegradation of the textile azo dye Red HE3B in yeast extract medium using a developed microbial consortium-SDS observed that decolorization performance was tremendously inhibited under shaking conditions (20% in 24 h) while complete dye removal was observed under microaerophilic conditions within only 1 h [59]. Hence, further degradation studies were carried out under microaerophilic conditions only.

### 3.3. Decolorization Parameters

The efficiency of biodegradation processes is greatly influenced by the operational parameters. Thus, culture medium pH, incubation temperature and dye concentration must be optimized for enhanced decolorization performance. The pH has a major effect on the rate of decolorization as the tolerance of bacteria to more acidic or alkaline pH values makes them suitable for biological treatment of dye wastewaters.

**Figure 5:**
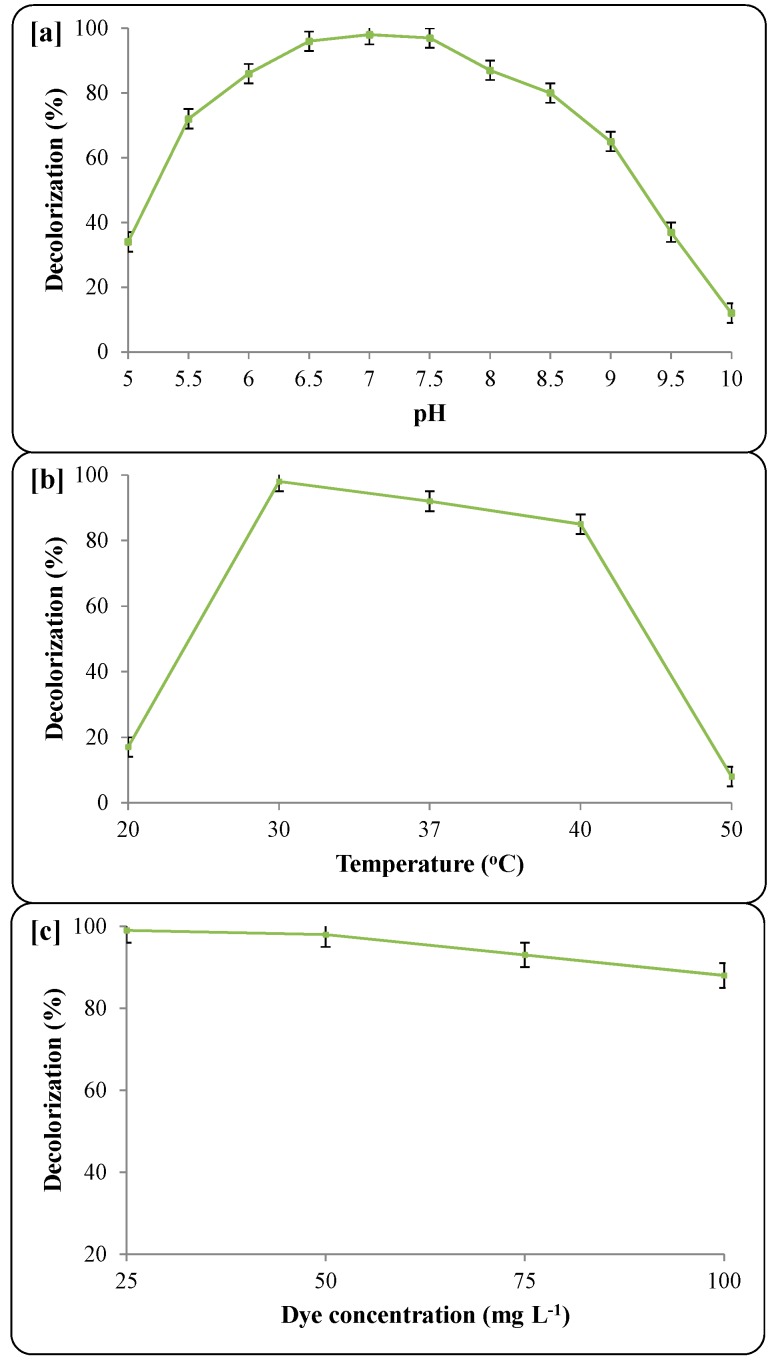
Effect of environmental parameters on decolorization of Trypan Blue (50 mg∙L^−1^) by microbial consortium; (**a**) initial pH of pre-enriched culture medium, (**b**) incubation temperature, (**c**) various dye concentration. Decolorization was measured after 24 h of incubation. Data points represents the mean of three independent replicates, standard error of mean is indicated by error bars.

The maximum decolorization of Trypan Blue (50 mg∙L^−1^) was observed at pH 7.0 and almost complete removal of dye was obtained within 24 h at 30 ± 0.2 °C temperature (Figure 5a). The decolorization performance of the microbial consortium was not much affected when the pH was lowered to 6.0 or raised to 8.5. However, at a more alkaline pH (10.0) the decolorization efficiency decreased and only 12% of the dye was removed within 24 h. The pH of the medium has been known to be responsible for the transport of dye across cell membranes and thus considered a rate limiting factor [60]. The results of our study indicated that, though maximum dye decolorization was observed at pH 7.0, the enriched microbial consortium could be used over a wide range of pH values (6–8.5) for dye removal. Balapure *et al.* observed a similar decolorization pattern for the dye Reactive Blue 160 by mixed cultures consisting of eight bacterial strains, where maximum dye removal was found at pH 7.0 and it decreased slightly within the pH 6–8 range [48].

The degradation of the dye Trypan Blue was monitored as a function of incubation temperature. To optimize this parameter, microbial consortium-mediated decolorization was investigated at different incubation temperatures, while keeping the other conditions the same (pH 7.0, Trypan Blue 50 mg∙L^−1^, microaerophilic incubation). The microbial consortium appears to work well at 30 ± 0.2 °C temperature; while lower and higher temperatures than the optimum slightly affected the decolorization rates. Almost 99% decolorization was observed at 30 ± 0.2 °C within 24 h; whereas an increase in temperature to 37 and 40 ± 0.2 °C resulted into 92 and 85% performance within the same time, respectively (Figure 5b). However, a higher increase (50 ± 0.2 °C) or a decrease (20 ± 0.2 °C) in incubation temperature tremendously inhibited the decolorization ability of the microbial consortium to 8% and 17%, respectively. The decrease in decolorization at 50 and 20 ± 0.2 °C might be because of cessation of microbial growth and/or thermal denaturation of protein and enzymes. However, significant decolorization over a wide temperature range points to further use of this consortium for large scale remediation of dyes at ambient temperatures. Our results corroborate the results obtained by Phugare *et al.* who observed maximum textile effluent decolorization by the bacterial consortium SDS at 30 °C followed by a slight change at higher and lower temperatures [61]. The degradation potential of bacteria is known to be associated with their growth and the enzyme activities at the respective temperatures [61].

Dye concentration is known to have an inverse influence on microbial survival and decolorization efficiency. Most dyes are visible in water at concentrations as low as 1 mg∙L^−1^ and textile effluents have concentrations ranging from 10 to 200 mg∙L^−1^ [62], making it thus essential to evaluate the maximum dye decolorization capacity of a microbial consortium for further biodegradation purposes. As shown in Figure 5c, the complete decolorization (99%) of Trypan Blue was observed for 25 and 50 mg∙L^−1^ dye concentrations within 24 h. The increase in dye concentration beyond 50 mg∙L^−1^ slightly affected decolorization performance as 93% and 88% removal was observed at 75 and 100 mg∙L^−1^ respectively. This decreased efficiency may be due to the toxicity of the dye and dye degradation metabolites formed at higher concentration [63]. Additionally, higher dye concentrations may also affect bacterial growth [64]. However, the ability of the microbial consortium to completely decolorize the highly sturdy and recalcitrant dye Trypan Blue (50 mg∙L^−1^) in agricultural waste medium demonstrated the importance of the present study. In summary, a microbial consortium grown in WB medium was found to be very effective for decolorization of Trypan Blue under optimum pH, temperature and dye concentrations, and thus could be used for large scale treatment of benzidine-based dyes.

### 3.4. Mineralization of Dye

The primary objective of biodegradation processes is to mineralize the dye into CO_2_ and H_2_O without generation of toxic intermediates. However, biodegradation of several azo dyes results into the formation of toxic metabolites such as aromatic amines which are known to be mutagenic and/or carcinogenic [27,65]. Thus, it is necessary to evaluate the degradation products for the presence of aromatic amines before being released into environmental sinks. Analysis of the decolorization process at regular time intervals suggests that 0.10 mM and 0.24 mM of aromatic amines were formed within the first 8 and 16 h, respectively (Table 1), but it was interesting to note that no amines were detected after the complete 24 h decolorization process. The presence of amines up to 16 h of incubation may be due to the initial reductive cleavage of azo bond by azoreductases under microaerophilic conditions. Azoreductases are known to utilize azo dyes as terminal electron acceptors in oxygen limiting conditions [66]. Further disappearance of the formed amines might be due to oxidative breakdown by laccase in presence of the remaining molecular oxygen. The role of laccase in oxidative cleavage of azo dyes is well documented [67]. This is very similar to what we have reported for decolorization of the azo dye Rubine GFL by a fungal-bacterial consortium, and thus underscores the usefulness of this consortium to decolorize benzidine-based azo dyes without generating aromatic amines.

**Table 1:** Decolorization of Trypan Blue (50 mg∙L^−1^) by a bacterial consortium and formation of aromatic amines at regular time of intervals.

Incubation Time (h)	Decolorization (%)	Amine Conc. (mM)
0	0 ± 0.0	n.d.
8	34 ± 1.5	0.10 ± 0.01
16	70 ± 2.0	0.24 ± 0.02
24	98 ± 1.0	n.d.

n.d. = Not detected. Values are mean of three experiments, ± Standard deviation for all the data.

Degradation of the dye Trypan Blue by a microbial consortium using optimized parameters was accompanied by mineralization analysis. The mineralization of a dye as represented by the important environmental parameters TOC and COD removal ratio were measured for the untreated dye and decolorized medium. Results of these parameters after complete decolorization (24 h) of dye by the microbial consortium revealed a decrease in the values of TOC and COD by 64% and 88%, respectively (Table 2). The COD removal by the microbial consortium was significant compared to solar energy-assisted photo-Fenton oxidation of Trypan Blue [14], which achieved a COD removal of 51.6%. The complete decolorization with high TOC and COD removal efficiency indicates the potential of the microbial consortium for decolorization as well as mineralization of Trypan Blue dye in WB medium. Thus, from an environmental as well as an economical point of view, the present microbial consortium could be used for decolorization of benzidine-based dyes at large scale since it reduced TOC and COD significantly while growing on agricultural waste WB under submerged conditions. To the best of our knowledge, there has been no use of wheat bran as a growth medium for biodegradation of benzidine-based dyes under submerged conditions.

**Table 2:** Environmental parameters of the untreated dye Trypan Blue (50 mg∙L^−1^) and after treatment with microbial consortium.

Environmental Parameters	Untreated Dye	After Decolorization (24 h)
TOC (mg∙L^−1^)	1642 ± 3.0	591 ± 4.0
COD (mg∙L^−1^)	1265 ± 5.0	152 ± 3.0
Color (%)	100 ± 0.0	1 ± 1.0

Values are mean of three experiments ± standard deviation (SD).

Since the cost of the medium plays a very important role in commercial applications of bioremediation methods, the real market price analysis of wheat bran with the most commonly used defined growth medium nutrient broth was carried out. The price of wheat bran on the world’s biggest online trading company Alibaba.com is US $154–162/metric ton; while that of nutrient broth is US $5000–20000/metric ton. Such a huge price difference between wheat bran and nutrient broth underlines the cost effectiveness of the present work to use it for large scale treatment of dye wastewaters. In addition, the remaining agricultural waste substrate after media preparation could be used as a low cost adsorbent for dyes and subsequent biodegradation in SSF-based processes [31].

### 3.5. Enzyme Studies

Decolorization of dyes usually occurs by enzymatic means. A recent book chapter by Telke *et al.* has shown that oxidative enzymes such as laccases, tyrosinase, veratryl alcohol oxidase from various microorganisms can oxidize dyes; while a few important reductive enzymes such as azoreductase, and NADH-DCIP reductase can perform a reductive breakdown of azo dyes [68]. In the present study, analysis of the enzymes responsible for biotransformation of the dye, *viz*. laccase, lignin peroxidase, azoreductase, and NADH-DCIP reductase revealed that microbial consortium produce these enzymes and thus the dye decolorization observed was likely to be enzymatically caused. Analysis of the oxidative enzyme laccase, tryrosinase and veratryl alcohol oxidase showed induction of 275%, 21% and 62%, respectively, after decolorization when compared with control cultures (Table 3). Similarly, the reductive enzymes azoreductase (500%) and NADH-DCIP reductase (264%) from decolorized medium also showed induction but at very high levels. Significantly higher activity of azoreductase and NADH-DCIP reductase in decolorized culture medium suggest a reductive cleavage of azo bonds. It is well known that azoreductase performs the initial reductive cleavage of azo bonds in microaerophilic conditions [69]. On the other hand, laccase was also found highly induced indicating the further oxidation of formed metabolites. The role of laccase in the oxidation of azo dyes and/or their degradation metabolites has been previously reported [67,70]. These obtained results showed high oxidative as well as reductive enzyme activities in decolorized dye culture medium as compared to control cultures. It suggests that enzyme activities were induced only if the microbial consortium was exposed to dye; indicating the combined action of all these enzymes is responsible for the decolorization and degradation of the dye Trypan Blue. Our results are consistent with the findings of Balapure *et al.*, in which induction in the activities of laccase, tyrosinase, azoreductase and NADH-DCIP reductase were observed in azo dye decolorized medium [71]. The formation of aromatic amines within the first few hours of the decolorization process and further disappearance after complete decolorization of dye thus indicates the initial reductive cleavage of dye by azoreductase and further oxidation of the formed amines by laccase.

**Table 3:** Enzyme activities in control cells and cells obtained after decolorization of dye Trypan Blue (50 mg∙L^−1^) by microbial consortium.

Enzymes	Control (0 h)	Test (After Decolorization for 24 h)
Laccase ^1^	0.4 ± 0.01	1.5 ±0.03 ***
Tyrosinase ^2^	0.8 ± 0.02	0.97 ±0.03 **
Veratryl alcohol oxidase ^3^	0.80 ± 0.02	1.3 ±0.05 ***
Azoreductase ^4^	0.6 ± 0.01	3.6 ±0.04 ***
NADH-DCIP reductase ^5^	12.2 ± 1.1	44.4 ±4.2 ***

Control = Enzyme extracted after enrichment of microbial consortium. Test = Enzymes extracted from the dye decolorized culture medium. Values are mean of three experiments ± standard error mean (SEM), significantly different from control cells at * *p* < 0.05, ** *p* < 0.01 and *** *p* < 0.001 by one-way analysis of variance (ANOVA) with Tukey Kramer comparison test. ^1^ µM of ABTS oxidized min^−1^ mL of enzyme^−1^ mg of protein^−1^; ^2^ µM of catechol oxidized min^−1^ mL of enzyme^−1^ mg of protein^−1^; ^3^ µM of veratryl alcohol oxidized min^−1^ mL of enzyme^−1^ mg of protein^−1^; ^4^ μM of Methyl red reduced min^−1^ mL of enzyme^−1^ mg of protein^−1^; ^5^ μM of DCIP reduced min^−1^ mL of enzyme^−1^ mg of protein^−1^.

### 3.6. Biodegradation Studies

**Figure 6:**
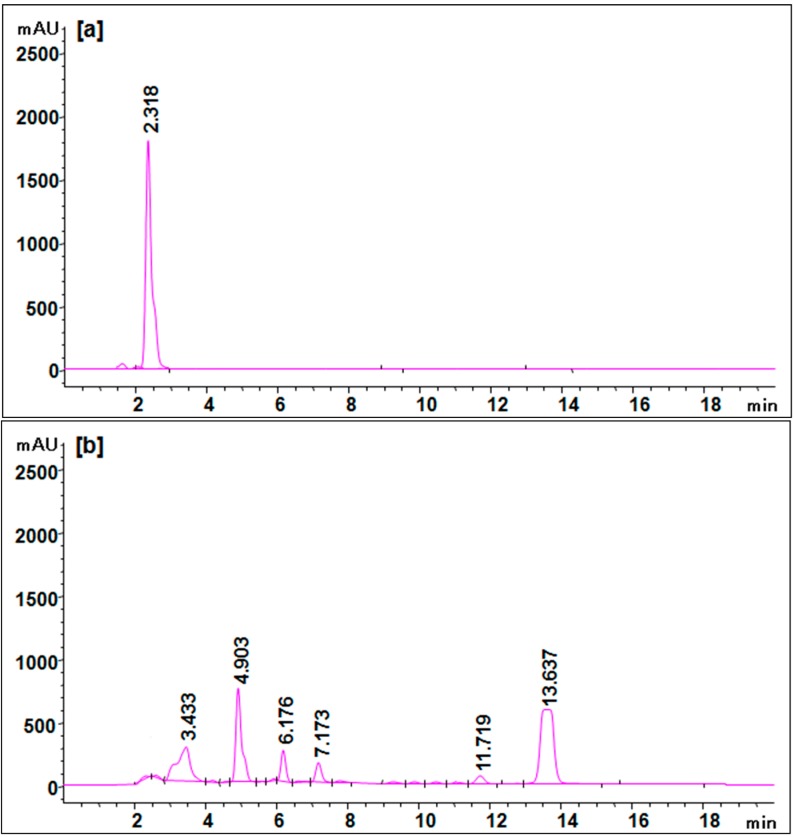
HPLC elution profile of (**a**) control dye Trypan Blue and (**b**) its degradation product at 210 nm.

In addition to mineralization studies, the biodegradation of Trypan Blue dye was monitored by HPLC, FTIR and GC-MS analysis as well. Figure 6 shows the HPLC chromatograms of control dye Trypan Blue and its degradation products after treatment with the microbial consortium. The HPLC profile of the control dye showed a single peak at a retention time of 2.318 min (Figure 6a). The microbial consortium-treated dye sample showed six peaks at different retention times which could correspond to the degradation metabolites. A total of two major peaks at retention time of 4.903 and 13.637 min as well as four minor peaks at 3.433, 6.176, 7.173 and 11.719 were observed (Figure 6b). As can be seen in the treated sample chromatogram, the appearance of six new peaks at various retention times indicates the total conversion of Trypan Blue dye into different metabolites. HPLC has been previously reported for the analysis of dye degradation metabolites [27,31,61]. Furthermore, in order to identify these metabolites by formed microbial consortium and to come up with a possible degradation pathway of Trypan Blue, we employed FTIR and GC-MS analyses.

**Figure 7:**
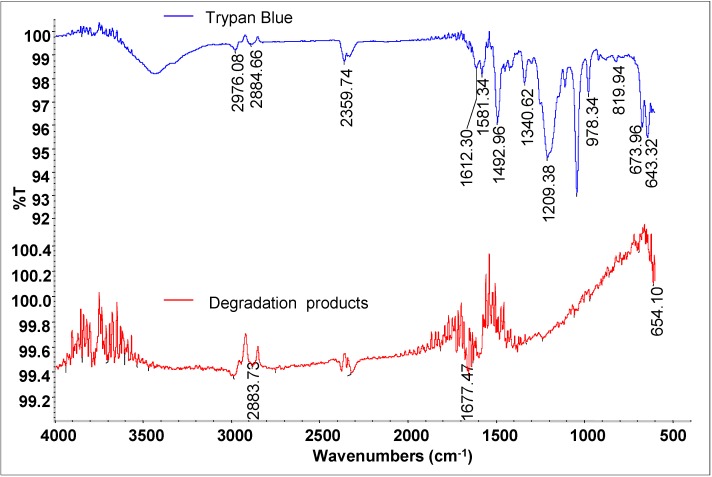
FTIR analysis of a Trypan Blue control and its degradation products obtained after 24 h treatment with the microbial consortium.

As shown in Figure 7, the FTIR spectra of control dye Trypan Blue showed vibrations at 2976.08 cm^−1^ (epoxides OC-H stretching), 2884.66 cm^−1^ (alkanes C-H stretching), 2359.74 cm^−1^ (amines NH^+^ stretching), 1612.30-1581.34 cm^−1^ (double azo bond –N=N stretching), 1492.96 cm^−1^ (nitrosamines N=O stretching), 1340.62 cm^−1^ (azides –N_3_ stretching), 1209.38 cm^−1^ (aliphatic amines C-N vibration), 819.94 cm^−1^ (benzene ring with three adjacent H atom C-H deformation) and the right part spectral region from 673.96 to 643.32 cm^−1^ (carbonated impurities).

After 24 h of treatment with microbial consortium, a different pattern of absorption peaks was observed for dye extracted products. The FTIR profile of dye decolorized products showed peaks at 2883.73 cm^−1^ (alkanes C-H stretching) and 1677.47 cm^−1^ (nitrites N=O stretching). Two peaks of untreated dye Trypan Blue at 1612.30 and 1581.34 cm^−1^ associated with the azo bonds were totally disappeared in decolorized products indicates the reductive cleavage of dye molecule. At the same time, number of peaks ranging from 810 to 750 cm^−1^ corresponding to benzene ring structure of Trypan Blue were also disappeared indicating the opening of dye benzene ring. These results evidently suggested reductive cleavage of azo bond of dye Trypan Blue by azoreductase and further degradation of formed metabolites by oxidative enzymes.

The intermediate metabolites formed were identified by GC-MS analysis and shown in Table 4. The GC-MS data supported the cleavage of azo bond leading to the formation of disodium 3,5-diamino-4-hydroxynaphthalene-2,7-disulfonate with a mass peak of 378. Another metabolite identified was naphthalen-1-ol, with a mass peak of 144. A possible pathway for the degradation of Trypan Blue was put forth on the basis of these identified products. As observed in the FTIR analysis of the control dye, peaks corresponding to high molecular weight compounds were not detected by GC-MS of the decolorized dye products, suggesting a complete breakdown of Trypan Blue into low molecular weight compounds.

**Table 4:** GC-Mass spectra of products obtained after degradation of Trypan Blue by microbial consortium.

Retention Time (min)	Mol. Weight (m/z)	Name of Metabolite	Mass Spectrum
23.152	378 (m/z = 380) (+2)	Disodium 3,5-diamino-4-hydroxynaphthalene-2,7-disulfonate [I]	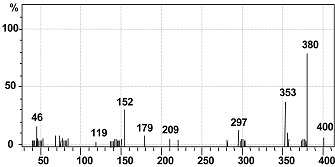
17.102	144 (m/z = 143) (−1)	Naphthalen-1-ol [II]	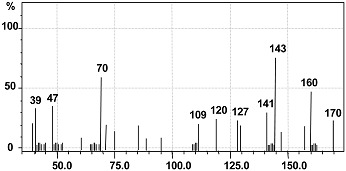

**Figure 8:**
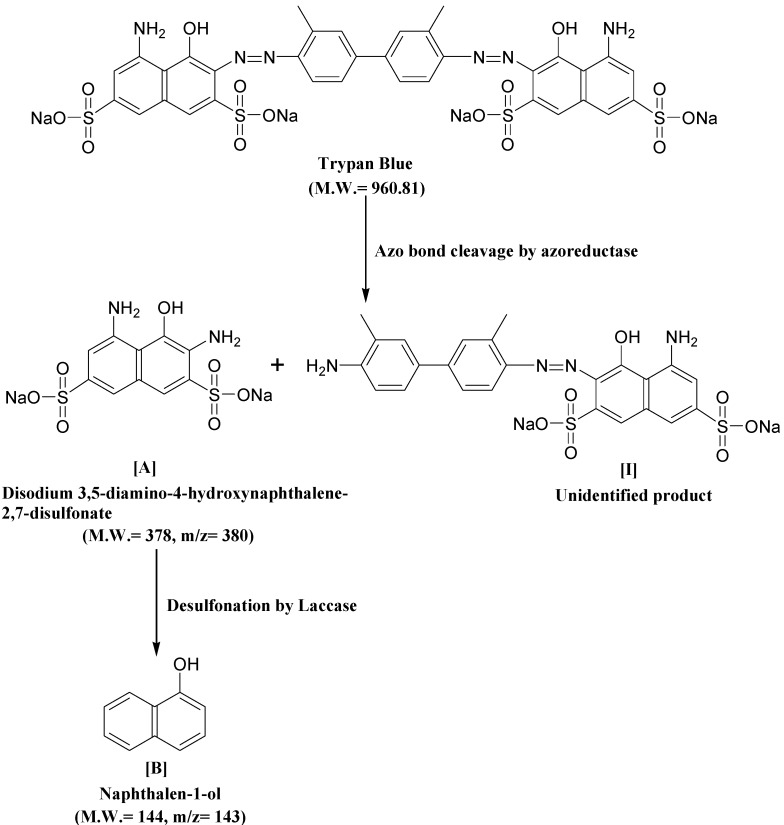
Possible degradation mechanism of Trypan Blue by the microbial consortium based on the GC-MS data and enzyme activities.

A possible mechanistic pathway for degradation of Trypan Blue by microbial consortium has been proposed and shown in Figure 8. As seen in figure, the initial step in bacterial degradation of azo dyes under microaerophilic condition involves the reductive cleavage of –N=N– bond facilitated by reductases leading to formation of low molecular weight colorless aromatic amines [72,73]. The above FTIR and GC-MS analysis data of the dye degradation products clearly confirms the initial reductive cleavage of Trypan Blue resulting into formation of an intermediate metabolite disodium 3,5-diamino-4-hydroxynaphthalene-2,7-disulfonate (A). This reductive cleavage is also supposed to generated the metabolite (I); however, further mineralization via the TCA cycle was anticipated due to the presence of tyrosinase activity (Table 3) and thus this was not detected by the GC-MS analysis. With the continuation of decolorization process, the metabolite-A was converted into a lower molecular weight compound naphthalen-1-ol (B) as final product via the oxidation by oxidative enzymes like laccase, tyrosinase or veratryl alcohol oxidase. The significant induction in the activity of laccase demonstrated their active role in oxidation of metabolite A.

### 3.7. Toxicity Studies

Analysis of the ecological and genetic impact of dyes on the growth of crop plants is of great importance as those are commonly consumed by humans. Results of phytotoxicity tests with *S. vulgare* and *P. mungo* showed that seeds exposed to untreated dye showed inhibition of germination by 80% and 70%, respectively, and also less shoot elongation (3.5 and 4.9 cm) and root (1.9 and 1.8 cm) lengths were observed (Table 5). On the other hand, seeds irrigated with dye degradation products showed 90% germination with good elongation of shoot (9.5 and 10.1 cm) and root (3.8 and 4.6 cm) lengths for both the *S. vulgare* and *P. mungo* plants, respectively. These parameters are almost similar to those of seeds irrigated with distilled water , where 100% germination, 9.8 and 10.2 cm shoot length and 4.1 and 4.8 cm root lengths were observed. These results are consistent with the findings of Kadam *et al.* [31], in which over 90% germination of both the plant seeds and considerable elongation of shoot and root lengths were observed for the products of degradation of the dye Solvent Red 5B by *Providensia staurti* strain *Ebt*SPG. Similarly, inhibition of germination, retarded shoot and root lengths in the presence of the dye Green HE4BD and their subsequent increase in dye degradation products by a developed bacterial consortium were reported [74]. Thus, phytotoxicity analysis indicated that the microbial consortium converted the toxic dye Trypan Blue into non-toxic metabolites.

**Table 5:** Phytotoxicity of Trypan Blue (50 mg L^−1^) and its degradation products extracted after degradation (24 h) by microbial consortium.

Samples	*S. vulgare*			*P. mungo*		
	Germination (%)	Shoot Length (cm)	Root Length (cm)	Germination (%)	Shoot Length (cm)	Root Length (cm)
Distilled water	100	9.8 ± 0.2	4.1 ± 0.2	100	10.2 ± 0.3	4.8 ± 0.3
Trypan Blue	20	3.5 ± 0.2 *	1.9 ± 0.1 *	30	4.9 ± 0.2 *	1.8 ± 0.2 *
Degradation products	90	9.5 ± 0.3	3.8 ± 0.2	90	10.1 ± 0.2	4.6 ± 0.2

Values are mean of three experiments, ± SEM, significantly different from control (seed germinated in distilled water) at * *p* < 0.1 by the one-way ANOVA with Tukey-Kramer multiple comparison test.

Acute toxicity tests with *D. magna* have been suggested as a first screening method for the assessment of any lethal toxicity of chemicals to mammals and humans [75]. The use of *D. magna* as a toxicity indicator for evaluation of textile effluent quality and also in the assessment of treatment plant efficiency was also documented [76]. In this study, an acute toxicity test with original (50 mg∙L^−1^), 25% and 50% Trypan Blue-containing medium showed complete mortality of *D. magna*, whereas 50% mortality was observed in 75% dye diluted culture medium (Table 6). Treatment of dye with the microbial consortium revealed the complete detoxification of dye degradation products as no *D. magna* mortality was found. These finding suggest that any release of untreated Trypan Blue dye poses a risk to the environment and to human health, whereas dye treatment with the microbial consortium could be used for complete detoxification.

**Table 6:** Mortality of *D. magna* exposed to 75% dilution of the culture supernatants containing Trypan Blue treated with microbial consortium.

Samples	Mortality (%)
Distilled water	0 ± 0.0
Trypan Blue	60 ± 3.0
Decolorized medium	0 ± 0.0

Values are mean of three experiments, ± SEM.

## 4. Conclusions

Agricultural waste wheat bran was utilized as a growth medium for the biodegradation of the benzidine-based carcinogenic dye Trypan Blue under submerged conditions. The ability of the microbial consortium to degrade the dye was mainly due to the activities of oxidative and reductive enzymes. The proposed pathway suggests biodegradation of Tryphan Blue. FTIR and HPLC analysis confirmed the degradation of the dye by the microbial consortium. Commonly used models in environmental risk assessment of dyes like phytotoxicity and acute toxicity tests confirmed the biotransformation of the dye into non-toxic forms. Clearly, these findings suggest that WB medium is a promising low cost alternative to defined growth medium for the bioremediation and detoxification of hazardous dyes.
